# Prenatal Diagnosis of Autosomal Dominant Polycystic Kidney Disease: Case Report

**DOI:** 10.3390/reports8020056

**Published:** 2025-04-23

**Authors:** Elitsa Gyokova, Eleonora Hristova-Atanasova, Elizabeth Odumosu, Antonia Andreeva

**Affiliations:** 1 Department of Obstetrics and Gynecology, Faculty of Medicine, Medical University-Pleven, 5800 Pleven, Bulgaria; 2 University Hospital “Saint Marina”-Pleven, 5800 Pleven, Bulgaria; 3 Department of Social Medicine and Public Health, Faculty of Public Health, Medical University of Plovdiv, 4002 Plovdiv, Bulgaria; 4 Faculty of Medicine, Medical University-Pleven, 5800 Pleven, Bulgaria; elizabeth.odumosu@outlook.com (E.O.); antonia.andreeva16@gmail.com (A.A.)

**Keywords:** autosomal dominant polycystic kidney disease (ADPKD), genetic kidney disease, prenatal diagnosis, genetic counseling, renal cysts

## Abstract

**Background and Clinical Significance:** Polycystic kidney disease (PKD) is the most common inherited kidney condition, affecting approximately 500,000 individuals in the US. It causes fluid-filled cysts to develop throughout the kidneys, leading to decreased kidney function. Autosomal dominant polycystic kidney disease (ADPKD) is the more prevalent form, subdivided into the *PKD1* and *PKD2* variants. *PKD1* variants typically result in more severe symptoms and an earlier need for dialysis compared to *PKD2.* A prenatal diagnosis of ADPKD is rare due to its late-onset manifestations, but early detection can be crucial for management and family counseling. **Case Presentation:** A 24-year-old woman, during her first pregnancy, presented for her first prenatal ultrasound at 22 + 2 weeks gestation. The ultrasound revealed an increased echogenicity of the renal parenchyma in the left kidney, with pelvic dystopia, while the right kidney appeared normal. Follow-up scans showed significant progression, with both kidneys exhibiting thinning parenchyma and cyst formation. The baby was delivered via an elective cesarean section at 38 weeks, and a postnatal ultrasound confirmed ADPKD. Genetic testing identified a heterozygous variant of the *PKD1* gene, *NM_001009944.3 (PKD1):c.9157G>A p.(Ala3053Thr)*, classified as likely pathogenic. The baby displayed electrolyte abnormalities but improved after a week of hospitalization. **Conclusions:** This case highlights an unusual early presentation of ADPKD in a fetus with no family history of the disease. A prenatal diagnosis through ultrasounds and genetic testing can aid in early detection and management, providing valuable information for family counseling. Regular monitoring and genetic identification are essential for managing ADPKD and improving patient outcomes.

## 1. Introduction and Clinical Significance

Polycystic kidney disease (PKD) is the most common inherited kidney condition, affecting approximately 500,000 individuals in the US alone [1]. It is a hereditary condition that causes fluid-filled sacs, known as cysts, to develop throughout the kidneys, progressively leading to decreased kidney function [2]. Depending on the type of genetic variant, an individual can inherit either the autosomal dominant polycystic kidney disease variant (ADPKD) or autosomal recessive polycystic kidney disease (ARPKD), affecting 1 in 400 to 1000 and 1 in 20,000 to 40,000 people, respectively [3]. ADPKD is subdivided, based on the variant, into *PKD1* and *PKD2*, accounting for 78% and 15% of the cases, respectively. Additionally, ADPKD can result from inheriting the variant from either parent or from a new variant in individuals without a known family history; this is known as non-inherited ADPKD [1].

The *PKD1* gene is located on chromosome 16, while the *PKD2* gene is located on chromosome 4, coding for polycystin 1 and polycystin 2, respectively. These two proteins work together to facilitate the communication between the cell and its surrounding environment to regulate cell functions, such as cell-to-cell communication, cell moving, and cell division, and aid the normal development of the kidneys. Primary cilia, produced by *PKD1* and *2*, are fingerlike projections lining the renal tubules that help to maintain the anatomy of the tubules [4,5]. Both *PKD1* and *PKD2* manifest the same symptoms; however, *PKD1* is usually more severe [2]. Individuals with *PKD1* variants tend to have larger kidneys, more severe kidney-related complications, and typically require dialysis earlier (around 55 years old) compared to those with *PKD2* variants, who experience milder symptoms and need dialysis much later, after the age of 70 [6].

Symptoms experienced by patients with ADPKD generally begin to manifest after the age of 20 years old and include pain in the lower back due to the growth of the cysts and kidneys overall, abdominal pain, frequent infections, and acute pain due to bleeding into the cyst or the passing or a calculus (kidney stones) [7]. Furthermore, signs include hematuria and hypertension as a result of the dysregulation of the renin–aldosterone angiotensin system (RAAS) [8]. As an outcome, patients with ADPKD experience decreased kidney function and, in some cases, progress to end-stage kidney failure. It accounts for approximately 10% of end-stage renal disease cases [9]. ADPKD and its related complications place a significant economic burden on healthcare systems. For example, dialysis costs the NHS around GBP 600 million per year, while a kidney transplant and follow-up care cost approximately GBP 2000 per person annually [10].

While the diagnosis of ADPKD in individuals is usually made through imaging techniques, including ultrasound (US), computer tomography (CT), magnetic resonance imaging (MRI), and DNA testing, the prenatal diagnosis of ADPKD represents clinical rarity due to the typical late-onset manifestations of the disease [11]. However, in some cases fetal morphology examinations via US may reveal an increased echogenicity of the renal parenchyma and enlarged kidneys with early cyst formation, raising the suspicion of ADPKD. This case analyzes the prenatal diagnosis of ADPKD during routine prenatal USs, emphasizing the importance of prenatal screening, genetic testing, and postnatal management.

Some cases of severe ADPKD can mimic ARPKD, with early clinical manifestations before the age of 15 years that can lead to significant perinatal morbidity and mortality, resembling the symptoms of severe ARPKD [12]. ADPKD may resemble ARPKD during prenatal assessments by exhibiting bigger kidneys, heightened cortical echogenicity, diminished corticomedullary differentiation, many medullary cysts, and reduced amniotic fluid levels. This atypical sonographic manifestation of ADPKD may be confused with ARPKD.

This case is notably important as it highlights an unusual early presentation of ADPKD in a fetus whose parents have no history of ADPKD, contributing to the limited literature on the prenatal diagnosis of ADPKD. It also underscores the importance of improving early detection and family counseling for ADPKD. The early detection of ADPKD through prenatal screening and genetic testing is crucial for appropriate postnatal management. This case underscores the significance of considering ADPKD in the differential diagnosis of renal abnormalities detected during routine prenatal ultrasound evaluations.

## 2. Case Presentation

### Patient Information

**Figure 1:**
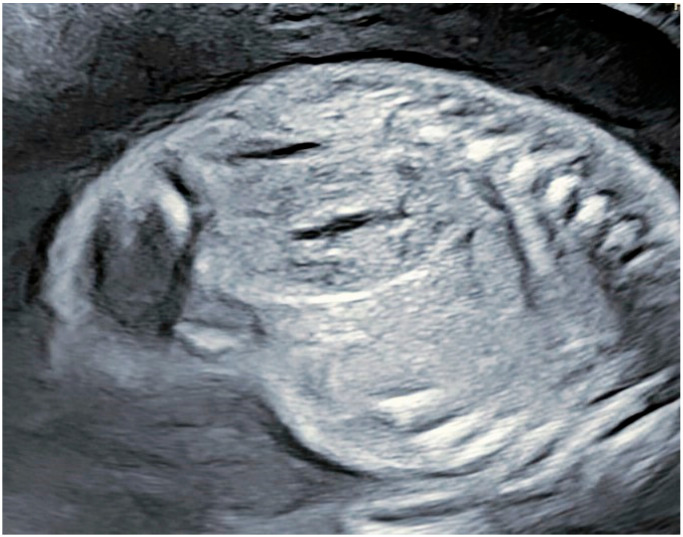
Appearance of right kidney at 22 g.w.—normal structure with normal amniotic fluid.

A 24-year-old woman, during her first pregnancy after a spontaneous conception, at 22 + 2 weeks gestation (g.w.), presented for her first prenatal ultrasound. She reported no complaints during the pregnancy and had an insignificant medical and family history. The fetal morphology examination revealed the unilaterally increased echogenicity of the renal parenchyma at the left kidney, with pelvic dystopia. The right kidney appeared with normal parenchyma for the gestational age without increased echogenicity or cysts described (Figure 1). The bladder and the amniotic fluid were within the normal ranges which was considered as a good prognostic factor. The were no other abnormalities detected on the fetal anatomy. The couple was given a favorable prognosis, reassured, and informed that the baby would require a postnatal ultrasound to assess renal function. Follow-up ultrasounds every 4 weeks were scheduled to monitor the fetal growth and amniotic fluid levels, as indicators of fetal kidney function.

**Figure 2:**
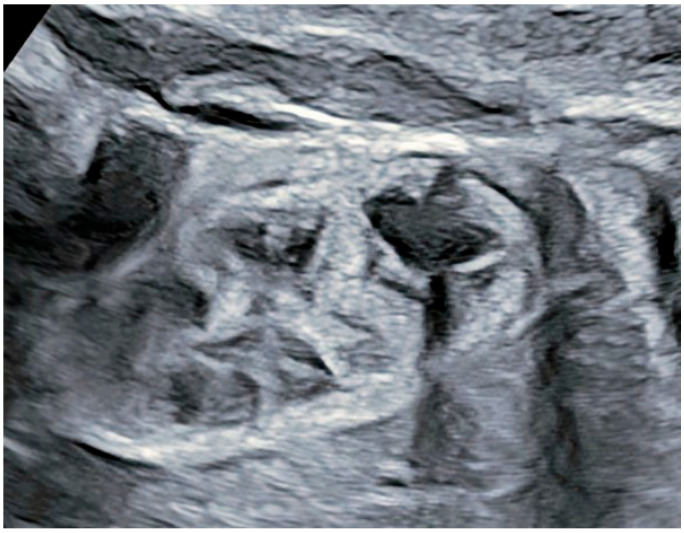
Appearance of right kidney at 34 + 1 g.w.—enlarged kidney with hyperechogenic contour with cyst-like collective structure.

At 34 + 1 g.w., the follow-up scan revealed significant progression in the anatomical appearance in both kidneys. The right kidney dimensions were enlarged on the 95th centile (Figure 2). Both kidneys had thinning of the parenchyma and dilated gathering spaces appearing as cysts. The contour of both kidneys was hyperechogenic, but no visibly dilated urethers were seen. The bladder appeared normal with a normal amniotic fluid level. The described findings were suggestive of ADPKD, which was later supported by a pediatric nephrologist. The couple decided to continue with their pregnancy, and the woman and fetus were placed under close monitoring during the pregnancy. A postnatal assessment of the renal function and genetic testing were scheduled to confirm the diagnosis and management, as per the late gestational age for performing invasive procedures.

**Figure 3:**
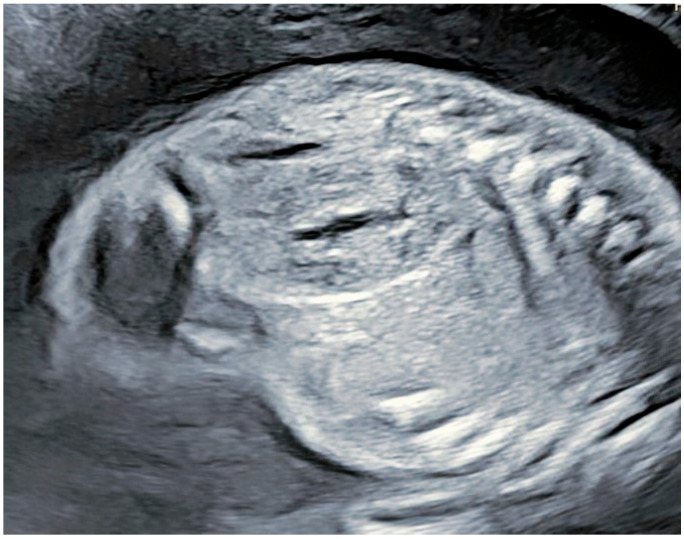
Appearance of right kidney at 36 + 1 g.w. (2 weeks later)—enlarged kidney with hyperechogenic contour with cyst-like collective structure.

At 38 weeks of gestation, the baby was delivered via an elective cesarean section. A postnatal US confirmed the prenatal suspicions of left kidney dystopia and ADPKD, exhibiting an increased echogenicity in both kidneys and multiple parenchymal cysts. Further testing revealed several electrolyte abnormalities, including hyponatremia and hypochloremia, hyperkalemia, and hyperbilirubinemia. After a week of hospitalization, the baby was discharged after displaying signs of physical and laboratory improvement. The family was given further guidance on long-term management and genetic counseling (Figure 3).

After the genetic analysis was completed by performing a DNA study of the single-nucleotide variants (SNVs) and copy number variation (CNV), the patient was reported as a heterozygote of the genetic type *NM_001009944.3 (PKD1):c.9157G>A p.(Ala3053Thr*). A Blueprint Genetics Cystic Kidney Disease Panel Plus Analysis was conducted, encompassing a sequencing analysis and copy number variation analysis. The panel focused on protein-coding exons, exon–intron junctions, and specific non-coding variations. It was utilized to identify single-nucleotide variants, minor insertions–deletions, and copy number changes, including single exon or bigger deletions and duplications. The diagnosis of ADPKD was confirmed, as a heterozygous variant of the *PKD1* gene was identified. The variant is classified as being likely pathogenic and is associated with cases of autosomal dominant polycystic kidney disease. Regular examinations are recommended in order to monitor the progression of the disease. Due to the prevalence of composite genotypes in numerous prenatal cases described in the literature, the patient’s sample was subjected to rigorous quality control measures, which included assessments for contamination and sample mix-ups. In addition, the presence of a second variant in either *PKD1* or *PKD2* was evaluated and rejected.

## 3. Discussion

The first prenatal diagnosis of ADPKD was documented by Zerres et al. in 1982, describing abnormally reflective, enlarged kidneys containing cysts [13]. Since then, numerous studies have been conducted on autosomal dominant polycystic kidney disease [14,15]. The median age of reaching kidney failure in ADPKD patients is 54 years, and patients are often asymptomatic before then [16]. Even so, a lot of cases demonstrate that it can be observed during the fetal period. An analysis of 83 documented cases of ADPKD that manifested in utero revealed that 67% of survivors had childhood hypertension, and 43% of them died during the first year of life [17,18].

In addition to our case, there was a rare presentation of ADPKD found manifesting prenatally [19]. The study reports on a family with two consecutive pregnancies affected by enlarged, cystic kidneys which were detected during the fetal development. This case highlights the complexity of genetic interactions in polycystic kidney disease and suggests that fetal manifestations resembling ARPKD can occur in the context of ADPKD due to combined variants in the *PKD1* gene. Therefore, an accurate diagnosis can only be made after childbirth. The case at hand pertains to parenchymal thinning in utero, which is an unusual presentation of ADPKD. Antenatal ADPKD is characterized by renal enlargement and heightened echogenicity [17]. Although uncommon, parenchymal thinning may indicate a more severe or fast-progressing phenotype and is underrepresented in the literature. This instance provides a valuable contribution to the range of prenatal ADPKD phenotypes. Comprehending the diverse phenotypes and possible indications of the illness severity might enhance management and therapy efforts. The additional investigation and documenting of these instances will enhance the knowledge of prenatal ADPKD and assist healthcare personnel in delivering more tailored care for afflicted individuals. When it comes to survival it depends on the lifestyle and the severity level of the deformations. ADPKD is characterized by an earlier onset, a higher number of cysts, and a faster progression to end-stage kidney disease (ESKD) [20]. Over time, the increase in both the number and size of cysts contributes to hypertension, bleeding, infections, discomfort, and pain [21]. Cyst growth plays a key role in the gradual loss of kidney function and tissue, occurring through direct mechanisms, such as parenchymal compression, or indirect pathways like fibrosis.

The prevalence of ADPKD is estimated to be below 5 in 10,000, which meets the European Union’s criteria for a rare disease [21]. A prenatal diagnosis and a good anamnesis are the keys for prevention and the early determination of fetuses with ADPKD.

Studies have explored the genetics of early-onset polycystic kidney disease (PKD), focusing on dual variants in *PKD1* and modifying genetic interactions. These studies highlight the complexity of PKD inheritance and the role of additional genetic factors in disease severity. A novel *PKD1* variant can modulate the disease severity in ADPKD, while additional variants can exacerbate disease severity [22]. The biallelic inheritance of hypomorphic *PKD1* variants is prevalent in early-onset cases, highlighting the importance of considering variant combinations in genetic analyses [23]. Comprehensive genetic testing is needed to fully understand the disease’s inheritance patterns and potential severity.

The recent literature has illuminated the prenatal manifestations of ADPKD and the obstacles associated with distinguishing it from ARPKD during fetal development. Advancements in prenatal imaging have enabled the earlier detection of acute kidney disease (AKD), which typically presents in adulthood. In utero, ADPKD may manifest as bilaterally enlarged, hyperechogenic kidneys with multiple cysts, often with normal amniotic fluid levels [24]. Very-early-onset (VEO) ADPKD, diagnosed prenatally or within the first 18 months of life, is associated with adverse clinical outcomes. Histological studies of fetal ADPKD kidneys reveal cystic dilatations, contrasting with the tubular involvement seen in adults [25]. Differentiating ADPKD from ARPKD prenatally can be challenging due to overlapping sonographic features. ARPKD typically presents with enlarged, echogenic kidneys, oligohydramnios, and an absent bladder visualization [26]. However, some ADPKD cases may mimic these findings, especially in the presence of homozygous *PKD1* variants [19]. Genetic testing becomes crucial for an accurate diagnosis, as variants in the *PKD1* or *PKD2* genes support the ADPKD diagnosis, while variations in the *PKHD1* gene indicate ARPKD.

The study by Audrézet et al. (2016) explores the genetic basis of early-onset ADPKD in prenatal cases [27]. The research analyzed 42 patients from 41 families with ADPKD, finding that 37.2% had additional *PKD1* or *PKD2* variations, a higher frequency than 14.4% in adult patients. The study emphasizes the need for comprehensive genetic screening in prenatal cases of ADPKD to understand the genetic interactions, which are crucial for accurate diagnosis, prognosis, and genetic counseling.

The early detection and management of ADPKD can help slow the progression of kidney damage and improve the quality of life for affected individuals. Genetic testing and regular monitoring are essential for individuals with a family history of ADPKD to ensure timely intervention and appropriate care.

The early diagnosis of ADPKD is crucial as it allows for prompt intervention and management, particularly in rapidly progressing cases which can lead to end-stage kidney disease in early childhood [28].

Upon early diagnosis, genetic counseling is essential to inform families about the autosomal dominant inheritance pattern and the associated risks to future offspring. The identification of a pathogenic variant in the index case can enable the cascade screening of at-risk family members and inform reproductive decision-making, including options such as a preimplantation genetic diagnosis (PGD) [29]. While there is no cure, several concrete steps can be taken early on to optimize outcomes and delay disease progression. The implementation of a kidney-friendly diet is imperative, as adequate nutrition is essential for supporting neurodevelopmental milestones and enabling the child to attain their full growth potential [30]. Breast-feeding the infant is encouraged as breast milk is known to have a reduced renal solute load because of its low phosphate and potassium levels [31]. A multidisciplinary team, including a pediatric nephrologist, geneticist, and potentially a pediatric urologist, should be engaged early on. In addition, regularly monitoring the disease progression is essential for the early identification of complications which may arise, including hypertension, abnormal heart valves, hepatic and pancreatic cysts, and recurrent urinary tract infections [32]. Timely medical and surgical management for these complications can improve the patient’s overall quality of life. Furthermore, some complications may present at birth; therefore, a prenatal diagnosis of the disease can allow for the facilitation of appropriate specialists to be present at the delivery to ensure necessary neonatal care. A common and life-threatening finding in neonates with PKD is severe respiratory failure secondary to pulmonary hypoplasia, often requiring intensive care management. Blood pressure should be assessed routinely beginning in infancy or early childhood, as hypertension may manifest early on and contributes significantly to morbidity [33,34]. Hypertension is a modifiable risk factor for disease progression. The initiation of angiotensin-converting enzyme (ACE) inhibitors or angiotensin receptor blockers (ARBs) may be warranted in pediatric patients with an elevated blood pressure or early signs of proteinuria [20].

Last but not least, the chronic nature of ADPKD and the demands of lifelong surveillance necessitate comprehensive family support. Early psychosocial intervention and developmental monitoring may benefit both the child and family system as the disease evolves [35].

To conclude, the early diagnosis of ADPKD provides a critical period for multidisciplinary care and nutritional support, mitigates complications, enhances quality of life, and improves the long-term outcomes of the affected children.

## 4. Conclusions

The prenatal diagnosis of ADPKD is a possible milestone and depends on the gestational age at presentation. The cyst visibility and the renal echogenicity, as well as the amniotic fluid volume and the existence of concomitant extra renal anomalies, facilitate the differentiation of bilateral polycystic kidneys observed on prenatal ultrasounds. Genetic identification might assist families and increase the clinical treatment of patients, potentially augmented by developing therapeutic choices. If ADPKD is identified in the perinatal period, this presents a rare but critical opportunity for early intervention, monitoring, and family planning support.

## Data Availability

The data presented in this study are available on request from the corresponding author. The data are not publicly available due to ethical restrictions.
